# Formal caregivers’ knowledge, attitudes, and challenges in end-of-life care for people with advanced dementia: a qualitative study in Chinese nursing home

**DOI:** 10.1186/s12912-025-03424-y

**Published:** 2025-07-01

**Authors:** Ye Ouyang, Chunyu Cheng, Qing Sun, Linbing Chen, Liping Wang

**Affiliations:** 1 Key Laboratory of Lifecycle Care for Chronic Diseases, School of Nursing, Guangdong Medical University, Dongguan, Guangdong Province 523808 China; 2 No. 1 Songshan Lake Science and Technology Industry Park, Dongguan, Guangdong 523808 People’s Republic of China; 3 Dongkeng Hospital Nursing of Dongguan, No.41, West Second Road, Dongkeng Town, Dongguan City, Guangdong Province 523808 China

**Keywords:** Formal carers, End of life care, China, Advanced dementia, Qualitative study

## Abstract

**Background:**

As frontline workers in caring for elderly people with dementia, the knowledge, attitudes, and working conditions of formal caregivers are crucial factors influencing the quality of end-of-life care. However, current studies focus on informal caregivers and lack of psychological discussion of formal caregivers. This study aims to investigate the knowledge and attitudes of formal carers implementing end of life care for patients with advanced dementia, along with the factors influencing their practice, to provide a foundation for enhancing the quality of end-of-life care for older adults.

**Methods:**

A qualitative descriptive study utilizing semi-structured interviews. Twelve formal carers with experience in caring for individuals with advanced dementia were recruited from Dongguan City, Guangdong Province, China. Participants were selected using purposive sampling, incorporating the principle of maximum variation. Qualitative data were collected through face-to-face interviews and analyzed using thematic analysis, with *NVivo14* software used for data organization.

**Results:**

Various negative and positive aspects, along with specific needs, were identified. Four key themes emerged from the interview analysis: (a) Comprehensive knowledge and attitudes of formal caregivers towards end-of-life care for dementia, (b) perceived occupational challenges, (c) motivational factors facilitating care delivery, and (d) specific needs related to caregiving.

**Conclusion:**

Formal carers of individuals with advanced dementia experience multiple pressures and challenges in their caregiving roles. Amid these difficulties, they develop self-coping strategies aligned with their role perceptions. Providing adequate care and support at all levels of the elderly end-of-life care system can alleviate caregiver stress and significantly enhance the overall quality of end of life care.

**Clinical trial number:**

Not applicable.

**Supplementary Information:**

The online version contains supplementary material available at 10.1186/s12912-025-03424-y.

## Background

Dementia, as described by the World Health Organization (WHO), is a progressive neurodegenerative syndrome marked by cognitive decline, behavioral disturbances, and functional impairments. It is currently the seventh leading cause of death among older adults worldwide. Dementia presents a significant global public health challenge [1] for aging populations. Core symptoms include memory impairment, disorientation, communication difficulties, and neuropsychiatric manifestations such as aggression and emotional apathy [2]. According to the “Chinese guidelines for the diagnosis and treatment of Alzheimer’s disease dementia (2020 edition),” dementia can be divided into five stages: (a) Preclinical stage. (b) Very early stage. (c) Early stage. (d) Mid-stage. (e) Late stage [3]. The special end-of-life care needs of people with dementia are reflected in the end stages of the disease: with patient-centered care at its core, respecting patient wishes through communication and shared decision-making; enhancing symptom management and comfort care, using assessment tools to identify pain and other discomforts, combining non-pharmacological and pharmacological interventions; setting nursing goals and plans early on, adjusting priorities as the disease progresses; ensuring continuous care, avoiding interruptions in referrals; providing psychosocial and mental support, valuing the calming effect of religious rituals for cognitively impaired patients; involving families throughout, covering education, bereavement support, and proxy decision-making support; multidisciplinary teams need specialized training to improve behavioral symptom management and ethical decision-making skills; avoiding overtreatment, adhering to individualized principles in nutrition and antibiotic use, balancing comfort with quality of life [4].

As of 15 March 2023, an estimated 55.2 million individuals worldwide were living with dementia, with over 60% residing in low- and middle-income countries. The global prevalence is expected to reach about 78 million by 2030 and 139 million by 2050, primarily due to population aging [5] in these regions. In high-income countries, end-of-life care frameworks for advanced dementia prioritize symptom management, quality of life, and family-centered decision-making, frequently facilitated through nursing home or hospice services [6]. For example, in the United States, 65% of patients with advanced dementia live in nursing homes [7] and address complex care needs by multidisciplinary teams [8].

In contrast, the dementia end-of-life care system in China remains underdeveloped. Although projections indicate that the proportion of individuals aged 65 and older will increase from 7% of the total population in 2002 to 14% by 2027 and 26% by 2050, with those aged 80 and above reaching 8% [9], the country continues to follow the ‘90–7 − 3 model’ [10], This model signifies that 90% of older individuals receive care at home, 7% receive care in community hospitals and health centers, and 3% are cared for in nursing homes by formal caregivers. Formal caregivers, classified as medical caregivers in China, include geriatric medical caregivers who primarily serve elderly populations [11]. These professionals, often referred to as paid carers, home health aides, or personal care workers, are employed by patients or their families to provide care in various long-term care settings, including residential facilities, continuing care hospitals, and private homes [12, 13].

From a global perspective, the role of formal caregivers (unlicensed nurses) in nursing homes varies significantly across countries and regions due to differences in aging policies and care systems. For example, in Japan, formal caregivers must undergo training as certified health care workers (over 450 h, including medical and psychological courses) [14]. In China, the training for formal caregivers focuses more on basic skills, with their core tasks encompassing medical assistance (such as vital sign monitoring), daily care, and psychological support, which fundamentally differ from “live-in nannies” who primarily provide domestic services ——, the latter typically not involving medical procedures or professional care theories [15], but lacking systematic knowledge of psychology or geriatric medicine, their tasks mainly involve daily care and low-risk medical assistance [16], still falling short of the specialized model of “medical-living integrated care” in developed countries [17]. Notably, in Chinese nursing homes, registered nurses and formal caregivers form a “collaborative-assignment” model: nurses are responsible for developing care plans and performing invasive procedures, while assigning tasks such as daily care and basic monitoring to caregivers, who participate in the care process under the guidance of nurses but lack independent decision-making authority. As a complementary force in the caregiving system, formal carers deliver comprehensive services throughout the aging process, assuming responsibilities traditionally held by family members. Their duties encompass assisting with daily activities such as bathing, dressing, transferring, eating, and toileting [18].

However, there are significant nursing issues among formal caregivers in nursing homes. Studies show that formal caregivers in nursing homes have poor knowledge of end-of-life care [19]. In China, the educational level of formal caregivers is generally low, with most having only primary or junior high school education, and a low proportion receiving systematic education on end-of-life care knowledge, which limits their systematic mastery of professional end-of-life care knowledge [20]. Some formal caregivers have a negative attitude toward end-of-life care, believing that general care is sufficient for the late stages [21]. Moreover, nursing homes lack sufficient caregivers, leading to an extreme mismatch between the number of patients and caregivers [22]. Coupled with the lack of corresponding subsidies for formal caregivers [23], this increases their caregiving burden. Additionally, the substantial physical and emotional demands of end-of-life care, along with societal underestimation of their role, make formal caregivers more prone to higher rates of depression [24]compared to the general population.

Although the essential role of formal carers is increasingly recognized, limited research has examined their knowledge levels, attitudes toward end of life care, and key influences on care provision, particularly in resource-constrained environments. A review of existing literature indicates that prior studies have predominantly focused on family carers, overlooking the unique challenges and pressures encountered by formal carers in professional settings. This research gap restricts efforts to enhance care quality and develop targeted policy measures. Moreover, Cultural aspects, including the strong emphasis on filial piety [25] and home-based care preferences [26], further complicate the integration of end of life care principles into China’s long-term care framework.

This study employs qualitative research methods to delve into the experiences of formal caregivers in Chinese nursing homes during the end-of-life care of patients with late-stage dementia. It aims to uncover key factors influencing the quality of end-of-life care, supplement knowledge about their current attitudes and identify modifiable factors affecting care quality. The goal is to provide information for formulating targeted interventions for formal caregivers and patients at the end-of-life care stage in nursing homes, and to promote policy reforms that support evidence-based end-of-life care models for elderly individuals with late-stage dementia. In the future, the findings of this study can be applied in nursing homes to implement relevant end-of-life interventions, thereby supporting the group of formal caregivers in Chinese nursing homes, reducing their caregiving burden, and improving the quality of end-of-life care.

## Methods

### Study design

This study employed a phenomenological qualitative research methodology [27], utilizing semi-structured interviews with the selected formal carers. Thematic analysis was conducted to analyze the interview data. The study adhered to the guidelines outlined in the Consolidated Standards for the Reporting of Qualitative Research (COREQ) [28], and the COREQ checklist has been included in the supporting information under Appendix S1.

### Participants

Eligible participants for this study were formal caregivers providing care for older adults with advanced dementia in Dongguan City, Guangdong Province, China. Participants were selected between 23 December 2024 and 23 February 2025 using a purposive sampling method that incorporated maximum variation [29] to ensure diversity in gender, age, education level, and length of service. The recruitment process involved obtaining a list of potential participants through the hospital nursing administration, distributing a written study brief to eligible carers, scheduling interviews, and formally including participants upon signing an informed consent form. The inclusion criteria were as follows: (a) formal caregivers in nursing home with direct experience of caring for patients with advanced dementia, (b) at least one year of service, and (c) willingness to participate. The sample size was determined based on the principle of information saturation [30], meaning recruitment ceased when no new themes emerged during data analysis. To ensure participant anonymity, numerical codes replaced names. No participants withdrew from the study.

### Data collection

A semi-structured interview outline with open-ended questions was developed by first author(YOY) based on a literature review and group discussion. A preliminary interview was conducted with one participant, and the interview outline was revised accordingly; the preliminary interview was included in the final study. Table 1 provides an overview of the semi-structured interview questions.

Qualitative data collection involved face-to-face personal interviews, which were recorded using professional recording equipment and mobile phone recording software. The interviews were conducted in a quiet meeting room within the care home, selected based on participant preferences, ensuring no interruptions. Prior to the interviews, a detailed explanation of the study’s purpose and significance was provided to ensure that participants were fully informed and voluntarily agreed to participate. Participants were notified that the interviews would be audio-recorded, their privacy would be protected, and they could withdraw at any time without facing any consequences. Additionally, participants were given the option to review the transcribed text of their interviews for verification. All participants signed an informed consent form and completed a brief demographic questionnaire to provide an initial understanding of their backgrounds. Field notes were taken by the researcher to capture non-verbal cues, including facial expressions, emotions, and body movements that were not explicitly mentioned in the interviews. Interview quotes used in the findings were translated into English.

A total of 12 formal caregivers (L1 to L12) participated in the interviews, which lasted between 17 and 50 min. All interview recordings and logs were securely stored as documents on a computer and a USB drive owned by the researcher.

**Table 1:** Outline of the semi-structured interview

Q1.	Can you describe the main manifestations of the patient’s late stages? What challenges do these performances pose to your caregiving? What do you think are the most important needs of patients at this stage?
Q2.	Have you received relevant training in care knowledge and skills before caring for patients? What do you think you need to be supplemented with education in caregiving?
Q3.	Do you feel the need for life-prolonging treatments for people with advanced dementia?
Q4.	What factors influence your attitudes and behaviors toward patients in your day-to-day care?
Q5.	In your actual care, have you ever encountered a conflict between the patient, the family, and the care provider? How do you make sense of these situations?
Q6.	Did the patient’s family have a end-of-life decision-making discussion with you about the patient’s condition? If so, what are the most important concerns for the family?
Q7.	Based on your experience in caregiving, what do you think can be done to improve the quality of care for patients?
Q8.	Is there anything else you would like to add to the care of people with advanced Alzheimer’s disease?

### Data analysis

The interview data were analyzed using Braun and Clarke’s thematic analysis method [31], which consists of six steps: (a) familiarization with the data, (b) generation of initial codes, (c) identification of themes, (d) thematic review, (e) definition and naming of themes, and (f) report writing. Coding was conducted with the assistance of NVivo14 software.

This study employs thematic analysis as the analytical method, primarily based on three considerations. First, its inductive nature aligns closely with the goals of phenomenological research —— by using open coding to directly extract naturally emerging themes from caregivers ‘interview texts, avoiding the segmentation of original experiences by prior theoretical frameworks, and authentically presenting the independent thinking of less educated caregivers due to their cultural background and work status. Second, the hierarchical structure of thematic analysis (primary themes → advanced themes) ensures a comprehensive capture of data details, preventing the omission of critical information. Finally, the transparency of this method’s process supports the credibility of the study, especially when dealing with the data characteristic of “the intertwining of everyday narratives and end-of-life situations,” ensuring that dimensions related to end-of-life care are extracted from the mixed narratives of formal caregivers, while also providing a traceable evidence base for subsequent practical recommendations (such as tiered training, process optimization).

Initially, necessary corrections were made to the interview transcripts to ensure accuracy without altering content. The transcripts were repeatedly reviewed to extract detailed information and minimize subjective bias. The text was then uploaded to NVivo14, where coding and data merging were performed. Themes were identified through group discussions and refined collaboratively by all researchers to enhance the validity of the findings. The analysis resulted in four key themes, which were supported by descriptive quotations to strengthen credibility and provide a clear representation of participants’ perspectives. All quotations were translated from Chinese by the researchers and subsequently reviewed by a professional language editing service.

This study ensured the rigor of qualitative research by adhering to the four dimensions of the trustworthiness framework: credibility, transferability, reliability, and confirmability. A three-stage verification mechanism was implemented during the interview process. First, a mock interview was conducted, and the questioning logic was refined before initiating formal interviews. Second, all interview recordings were transcribed verbatim within 24 h, and interview reflections were documented. Finally, the research team cross-referenced the audio recordings with the transcribed material to ensure 100% content consistency.

## Results

### Participant characteristic

A total of 12 formal carers participated in the study, comprising 10 females and two males. Among them, 10 had completed secondary education, while two had attained tertiary education. Their experience in caring for older adults with dementia ranged from 2 to 10 years, with all participants providing care for 12 h per day. The demographic characteristics of the participants are presented in Table 2.

**Table 2:** Sociodemographic characteristics of participants (*N* = 12)

Characteristic	Categories	Frequency(*N* = 12)
Gender	Male	2
	Female	10
Age (years)	20–35	1
	36–50	5
	51–65	6
Education Level	Middle school	7
	High school	3
	Junior college or above	2
Marriage	Unmarried	1
	Married	11
Seniority (years)	2–5	8
	6–9	3
	10 and above	1

### Themes

From the interview data, four themes and nine sub-themes were extracted. These include: (a) Comprehensive knowledge and attitudes of formal caregivers towards end-of-life care for dementia, (b) perceived work threats experienced by formal caregivers, (c) motivations for formal caregivers to improve care quality, and (d) specific care needs of formal caregivers. Table 3 summarizes the themes. To enhance the reliability of the research findings, representative statements were included, with each formal caregiver identified by a designated letter (e.g., L).

**Table 3:** Topics related to knowledge, attitudes and challenges of formal caregivers in nursing homes for patients with advanced dementia in China

Themes	Sub-themes
Comprehensive knowledge and attitudes of formal caregivers towards end-of-life care for dementia	Dementia routine end-of-life care issues
	Formal carer role perception
Formal carers’ perceived work-related threats	Individual Career Dilemmas
	Interpersonal dilemmas
	Resource Management Dilemma
Formal carers’ motivation for enhancing care quality	Internal: due diligence behavior and positive mindset
	External: solidarity and external support
Formal carers’ specific care needs	Technical manpower requirements
	Psychological needs

### Theme 1: comprehensive knowledge and attitudes of formal caregivers towards end-of-life care for dementia

The knowledge and attitudes of formal caregivers are crucial in determining the quality of care for individuals with Alzheimer’s disease. Especially in end-of-life care settings, a comprehensive understanding of the full behavioral patterns during the terminal phase, cognitive decline patterns, and changes in physical and mental needs is essential. This forms the core foundation for providing precise symptom management, maintaining dignity, and offering emotional support, directly impacting the comfort and humanistic value of end-of-life care. Additionally, awareness of dementia-related symptoms enhances formal carers’ perception of their roles and responsibilities. This theme is categorized into two sub-themes: (a) Dementia routine end-of-life care issues and (b) perception of the formal carer role.

### Dementia routine end-of-life care issues

#### The status of caregivers under attack

Throughout the provision of formal care for older adults with dementia, all carers reported encountering aggressive behavior. Such behavior is widespread and severe, posing risks to both the psychological and physical well-being of caregivers. Manifestations include verbal abuse and physical aggression. Some participants described in detail the persistent verbal hostility they received while caring for patients with advanced dementia at the end of their lives, which posed a challenge to the care work.

It is just swearing, swearing badly. (L11)

There is a guy in the VIP over there… Cursing people there all day… He does not even change your nappy at night. (L2)

In addition to verbal attacks, some patients with advanced dementia also exhibited physical aggression, directly harming the caregivers. One participant expressed her emotional agitation during the confessions, accompanied by sighs of helplessness and appropriate body movements. She said:

Punching people… One time our colleague was just punched by him (referring to the brow bone)… some time ago he was punched again, here on the forehead… Very hard. (L2).

However, for formal caregivers, aggressive behavior may serve as a compensation for the patient’s communication difficulties. Some participants shared that some patients would alter their behavior due to dissatisfaction with the caregiver’s arrangements. They need to recognize this as a signal of discomfort and pain from the patient. Caregivers must be acutely aware of this essence, avoid passive avoidance, actively decode the underlying needs behind the behavior, and respond positively to the patient’s expressions of distress:

…We told him to go to bed, and he immediately stopped, so he came with punches and kicks. (L9)

### Memory care challenges

Memory loss is a prevalent issue among older adults, characterized by the immediate forgetting of recent events. This rapid decline in short-term memory creates significant challenges for caregivers. One participant described this difficulty:

He just simply does not remember what he did at all… What he does one second, he just does not know the next… (L10)

According to the formal caregivers, they are often forced to repeat their efforts when planning meals and arranging accommodation for the patients with dementia because of their forgetting of immediate information. Such repetitive efforts seriously consume their time and energy, disrupt the preset care rhythm, and lead to a significant loss of care efficiency. For example:

He is just eaten, and he says he has not eaten… He is got some things… He is put them away himself… He’ll say the next day that his things are gone again. (L3)

Medication management is particularly challenging, as individuals may forget whether they have taken their prescribed doses, leading to repeated requests for medication. This not only complicates caregivers’ supervision efforts but also increases the risk of inappropriate medication use. The constant need for vigilance places caregivers under prolonged mental stress, leading to physical and emotional exhaustion, and increasing the likelihood of burnout.

### Support for homesickness

From the perspectives of psychology and neuroscience, as cognitive abilities decline, the social networks of elderly individuals with dementia gradually narrow from diverse relationships to a strong dependence on core family members. Although memory impairments make it difficult for seniors to express their needs fully in words, caregivers can still recognize their deep longing for familial connections through non-verbal signals such as physical gestures and emotional responses. Especially during the final stages, when life approaches an irreversible endpoint, this emotional need becomes even more intense due to the dual collapse of cognition and bodily functions. The caregivers involved in the study consistently displayed profound empathy and full understanding of late-stage terminally ill patients in their reflections. However, some participants also expressed in subsequent interviews that they do not wish to spend their end-of-life period alone like these patients:

…It is homesickness for loved ones… (L12)

…Each one wants to be at home. (L9)

Their today is our tomorrow… I see them like this… and I want it to be different in the future (L10)

### Formal carer role perception

In China, the role of formal caregivers exhibits multidimensional complexity. They not only invest substantial emotional labor —— such as patiently soothing the fears and anxieties of terminally ill patients, offering them comfort despite their inability to understand, but also carefully guiding family members through their grief and helplessness; they must also engage in high-intensity physical labor, like frequently assisting with patient repositioning and daily care tasks. However, within the current social cognitive framework, this profession faces severe underestimation and dual stigmatization. It is seen as an “undignified” job, suffering from neglect at the societal level, and is in a state of value degradation within social structures, making it difficult to earn the respect and recognition it deserves. A caregiver expressed their inner feelings thus:

That’s the lowest level of work, it is the dirtiest and the most tiring. (L3)

Additionally, caregivers themselves often regard their profession as inferior and struggle to gain understanding or support from their families. But it had little effect:

…Being here in this industry, belonging to the kind of the lowest… family members are… looking down on our kind… cross-eyed kind of feeling. (L11)

This reflects the low recognition of elderly care professions in society. Common social biases hinder caregivers from receiving the respect and acknowledgment they deserve. This cognitive bias about the value of the profession places additional psychological pressure on formal caregivers, who already bear special emotional and physical burdens. It also further impacts the quality of end-of-life care services and industry development:

I think this profession… is hard, aggravating, dirty, and tiring… It is quite a hard thing to do. (L10)

Despite the challenging work environment, some caregivers deeply understand that in the specialized field of end-of-life care for the elderly, high psychological resilience is key to handling complex situations. When facing the physical and mental changes of terminally ill patients and the grief and misunderstanding of their families, besides professional nursing skills, emotional regulation and mental strength are particularly crucial for those who persistently stay in this emotionally charged domain. Caregivers expressed this awareness:

…A good mindset is necessary. If you are not optimistic, you will feel really bad doing this job. Many of our colleagues struggle emotionally. (L9)

…People in this profession are remarkable… One must be very strong internally… Complaining about everything is unnecessary if you have chosen to do it. (L10)

This cognitive framework serves as the psychological foundation for professional resilience, enabling caregivers to maintain emotional stability under work pressure. This perspective reinforces their resolve to serve the elderly, particularly focusing on the well-being of seniors in their final stages. Their professional satisfaction primarily stems from witnessing the actual peace and comfort that care recipients achieve during end-of-life care:

We are supposed to be here to look after the elderly… I do not care which elderly person I am looking after, I hope… It is really helpful to him… If he is at ease in his heart, we are at ease in our hearts. If he feels comfortable, we feel comfortable too. (L9)

This reflects the deep-seated recognition of the value of their work among caregivers, aligning with China’s elderly care philosophy that emphasizes the needs of end-of-life care for seniors. Additionally, it demonstrates their pursuit of self-worth in their professional roles, which is consistent with Maslow’s hierarchy of needs theory, where “self-actualization” serves as the driving force for their career —— meaning that they achieve a unity of personal value and professional mission by ensuring the peace of the elderly during their final moments.

### Theme 2: negative work threats by formal carers

In China’s nursing homes, formal caregivers face multiple compounded challenges in their end-of-life care roles: the heavy workload of daily care is further magnified in terminal settings, while the relatively low compensation system fails to match the professional emotional labor invested in end-of-life care. Moreover, the lack of standardized end-of-life care guidelines and tiered management systems in nursing homes leads caregivers to rely heavily on experience when dealing with complex situations such as fluctuations in patients’ terminal symptoms and family members ‘fingerprints, which in turn fosters occupational burnout and value confusion. These structural contradictions not only erode caregivers’ professional identity in end-of-life care but also hinder the sustainable development of the end-of-life care sector within China’s long-term care system from both the perspectives of talent retention and service quality. This theme is categorized into three sub-themes: (a) personal career dilemma, (b) interpersonal dilemma, and (c) resource management dilemma.

### Individual career dilemmas

#### Negative work emotions

High-intensity and continuous nursing work imposes significant physical and mental stress on formal caregivers. Emotionally, almost all participants reported experiencing negative emotions such as aggression, fatigue, and impatience in end-of-life care. Despite receiving training in the principle of “neutrality at work,” managing emotions in actual caregiving situations remains a considerable challenge. One caregiver with five years of experience noted:

People say they do not bring emotions into work, that’s just theory… It is impossible not to bring it in real life. (L1)

Regarding labor intensity, structural human shortages have led to widespread work overload. In qualitative data, the recurring theme of “work overload” is particularly strong, with almost all caregivers reporting that their responsibility to care for multiple individuals exceeds their assigned workload. Moreover, due to the nature of nursing homes in China, formal caregivers often have to manage eight patients with different disease stages, which not only intensifies physical fatigue but also triggers emotional exhaustion, leading to severe work-related burnout:

Especially when you work night shifts… One person is in charge of ten or twenty elderly people… It is too busy. (L2)

It is very hard, too much care. (L7)

Sometimes it is just quite busy, it is non-stop. (L8)

Formal caregivers often experience significant caregiving stress, especially when helping older adults with heavier weights. Because of the physical decline and reduced ability to cooperate in end-of-life patients, caregivers often have to bear high physical loads in unconventional positions. Participants described the challenges these tasks present:

… 100s and 200s of pounds… Those are very tiring. (L5)

Some are so heavy, some have hemiplegic feet on one side and hemiplegic hands on the other. You dress him in a death grip, you go to give him a shower, a dress… You have a hard time. (L7)

### Interpersonal dilemmas

In the context of end-of-life care, the interpersonal challenges faced by individuals with negative work experiences exhibit particular complexity: caregivers not only have to deal with the anxiety of patients ‘families due to health changes in the late stages of illness but also frequently encounter their irrational expectations of routine care —— These expectations stem from a cognitive bias regarding the goals of end-of-life care, leading family members to over-analyze nursing details. When the patient’s condition falls short of expectations, caregivers often become outlets for family members’ frustrations and are further “instrumentalized.“:

He always seems to be uncomfortable with you people caring for him… He is always telling him what to do… Saying this and that… (L3)

Sometimes you do something for him, and then he has to say how well you did it. (L8)

Due to the differences in personal ability, working style and personality, it is difficult to achieve absolute fairness in workload distribution among nursing teams, which leads to cognitive conflicts among colleagues about responsibility transfer and ability bias, and has a negative impact on the collaboration efficiency of end-of-life care teams:

… There are individuals who he is not so good at talking to, and that creates some minor conflicts. (L1)

… We then have verbal fights… There was a little quarrel. (L2)

### Resource management dilemma

#### Lack of a Unified Management Approach

In this care home, formal carers were supervised by nurses; however, within the hierarchical aged care system, they encountered structural challenges due to inconsistent normative authority. There are differences in the management concepts and implementation standards of nurses in end-of-life care, which leads to the lack of uniformity in management practice and thus reduces the standard of nursing procedures. Caregivers frequently faced an ‘implementation paradox,’ wherein following the directives of one nurse led to accusations of non-compliance from another. This inconsistency caused confusion and frustration, as expressed by one participant:

There are so many leaders, so many disagreements… It makes the carer just confused, and they do not know what to do… (L1)

Caregivers noted that these recurring contradictions not only created cognitive dissonance but also contributed to workplace dissatisfaction through a ‘blame-shifting’ mechanism, where management inconsistencies were transferred as individual responsibility. Over time, such issues could negatively affect both caregiver morale and the overall quality of the elderly care system.

#### Human resource scarcity

In the field of end-of-life care, the imbalance between caregivers and patients is particularly prominent. When formal caregivers face a number of patients exceeding their capacity, they are forced to compress individual care times. For example, the intervals for critical operations like turning a dying patient are extended beyond standard times, increasing the risk of complications such as bedsores and aspiration pneumonia. Two participants shared insights on this issue:

One nurse may have to take care of eight old people. When the number is large, it is naturally not so good to satisfy them… To turn over… to wipe their bodies, some old people may need to apply medicine… and to urinate and defecate, which all take a lot of time. (L1)

Because there are so many people, you have to rush from one thing to another… If there are so few people, one person can do it carefully, clean up all the people up and down, or you can do whatever you need to do for him (L5)

### Theme 3: formal carer care for motivation

In the context of end-of-life care, positive influences play a crucial role in maintaining high-quality end-of-life care services. Adequate motivation not only enhances the involvement and professional satisfaction of caregivers in end-of-life care but also contributes to the overall stability of these services. These factors serve as support mechanisms, which are essential for the sustainable development of the end-of-life care component within China’s long-term care service system. They form the foundation for ensuring continuous improvement in care quality. This theme is categorized into two sub-themes: (a) internal factors, including responsible behavior and a positive mindset, and (b) external factors, encompassing solidarity and external support.

### Internal: due diligence behavior and positive mindset

Formal caregivers, as the primary providers of end-of-life care for the elderly, play a crucial role in ensuring the quality of life for seniors at the end of their lives. Their daily care not only addresses the physical, psychological, and social needs of those being cared for but also serves as a key indicator for evaluating the quality of end-of-life care in nursing homes. Most formal caregivers exhibit a strong sense of professional responsibility and internalize the ethical principle of “elder-centered care,” committing to providing comprehensive end-of-life care during working hours and meeting the reasonable needs of patients with advanced dementia:

…As a carer, we should certainly do our part to take good care of him… Try to fulfill him as far as we can. (L1)

Providing high quality end-of-life care requires a variety of skills, and caregivers emphasize that in addition to a strong sense of responsibility, patience and attention to the physical and mental characteristics of patients at the end of life are also key to providing detailed and comprehensive care:

Strong sense of responsibility… Good care… Have to be caring… Have to be a little bit careful. (L4)

… (For the elderly) you have to be really attentive, very attentive and have the patience to take care of him, really. (L7)

In the context of end-of-life care, a positive mindset is a continuous driving force for responsible nursing, as formal caregivers often project their forward-looking roles to establish psychological connections between the current experiences of terminally ill patients and their own potential future aging scenarios. Some caregivers report that they are influenced by personal family experiences in their end-of-life care work, especially the grief over losing parents, which enhances their ability to empathize with the emotional needs of terminally ill patients and their families:

Their today may be our tomorrow. (L10)

Because my mum just left too… and then I feel indebted to the elderly… with that guilt and indebtedness, that’s why I come to take care of these elderly people. (L9)

This sense of intergenerational empathy not only strengthens professional identity but also fosters a sustained sense of responsibility, enhancing caregivers’ commitment to their work. In addition, a positive attitude promotes reflective practice in the complex situation of end-of-life care and encourages caregivers to continuously improve the nursing methods for the special needs of dying patients. Through this continuous improvement attitude, caregivers build a virtuous cycle of “practice-reflection-optimization” in end-of-life care practice, carers establish a virtuous cycle of ‘practice-reflection-optimization,’ as highlighted by one participant:

We optimize ourselves… Doing it wrong… and then we correct and improve. (L9)

### External: solidarity and external support

Formal carers’ service efficacy is influenced not only by individual professional competence but also by teamwork effectiveness and external recognition. In the end-of-life care team, there is a complementary division of labor between formal caregivers and nurses, working together to address the complex needs of end-of-life patients, including physical and mental care, pain management, and support for family members’ grief. When responding to inquiries from the families of terminally ill patients, caregivers use an “information filtering” strategy, ensuring that professional information is accurately conveyed while also taking into account the emotional state of the family members, thus maintaining clear professional boundaries. This approach was described by carers as follows:

If he does have a problem… I would say that’s a situation where you’d have to ask one of our more specialized nurse practitioners. (L1)

He asked me… I said you talk to the nurse. (L7)

Additionally, recognition and understanding from family members serve as a vital source of social support. Some caregivers have noted that family members recognize their efforts in daily care, medical assistance, and psychological support for terminally ill patients. They deeply understand the complexity and challenges of end-of-life care, particularly in managing symptom fluctuations in the late stages of illness and supporting family grief. This support creates a positive psychological environment, effectively reduces occupational stress, and strengthens the caregiver’s professional identity and sense of belonging:

… Families are quite OK, and they understand that we work hard in this profession. (L12)

Furthermore, external support systems at multiple levels and across various dimensions play a crucial role in enhancing caregiving effectiveness. Almost all caregivers reported that the hospital has tailored systematic training programs to meet the specific needs of end-of-life care, continuously enhancing their professional capabilities. These trainings cover a wide range of topics, closely aligned with end-of-life care practices, building a comprehensive and specialized knowledge framework for end-of-life care, ensuring that caregivers can effectively address the complex challenges of caring for terminally ill patients:

A lot of training… Taught us so much, learned so much. (L6)

Basically, all the training… Because they are all more specialized. (L8)

The training is very comprehensive. (L10)

Additionally, an efficient material replenishment system serves as a fundamental safeguard for caregiving services. When material shortages occur, this system is activated promptly, minimizing the risk of service interruptions and maintaining stability in care quality:

Which place (needs goods) is not working… He immediately… Remedied it. (L4)

### Theme 4: formal carer care needs

The interviews indicated that, given the limitations of the current elderly end-of-life care system, the needs of formal carers are both diverse and individualized. This theme is categorized into two sub-themes: (a) technical manpower needs and (b) psychological needs.

### Technical manpower requirements

In the end-of-life care environment, formal caregivers frequently encounter technical challenges and physical stress (such as hand fatigue and back injuries) due to the lack of standardized guidelines and specialized assistive devices for end-of-life care. Therefore, some formal caregivers urgently hope that nursing home will provide specialized end-of-life care training and introduce assistive devices to reduce physical burdens and improve work efficiency, they say:

… There does not seem to be an assistive thing that does not teach… What is the best way for us to lift the elderly without damaging the carer’s back… To teach us more… Something to apply for assistance inside the hospital. (L5)

In addition, almost all participants reported dissatisfaction with the allocation of human resources. The current shortage of staff has led to a continuous increase in workload, and the complex care needs of terminally ill patients have been further exacerbated by this shortage, which negatively impacts the quality of end-of-life care services. Many caregivers emphasized the need for more staff and optimized scheduling to reduce work pressure and ensure the quality of care for terminally ill patients:

To have better quality… Need to reduce the number of carers. (L1)

More people would be better, we would not have to work so hard. (L7)

Without caring for so many elderly people, (we) spend more time with them. (L8).

These concerns highlight the critical importance of properly deploying staff in end-of-life care settings —— By scientifically allocating human resources, caregivers can devote more time to emotional support and personalized care for terminally ill patients, rather than being bogged down in basic nursing procedures. This human resource optimization directly points to enhancing the core value of elderly end-of-life care services.

### Psychological needs

The challenges faced by formal caregivers in the workplace stem not only from physical labor but also from the undervaluation of emotional labor and a lack of social recognition. Due to societal limitations in understanding end-of-life care for formal caregivers, their psychological needs have long been overlooked. Especially in high-frequency scenarios involving death, the repetitive emotional strain and the disconnect with social acknowledgment exacerbate their urgent need for workplace respect and psychological support. Caregivers have expressed their psychological expectations:

They never ask you about your hot, cold, warm problems, and they do not care if there is a family reason… Will it affect work? They never ask. (L1).

To come and care for us from time to time… Greet us… (L1)

Leaders can understand a little more; families understand us a little more. (L2)

These statements reflect the need for more empathy and recognition from management and families of dying patients to formal carers. Addressing these issues can improve occupational satisfaction and emotional health in end-of-life care work, contributing to a more supportive environment for end-of-life care.

## Discussion

This study focuses on the end-of-life care scenarios for patients with advanced dementia, identifying the multidimensional challenges and underlying motivational mechanisms faced by formal caregivers in this process. Through in-depth interviews and qualitative data analysis, four core dimensions of formal caregivers’ practices in end-of-life care for patients with advanced dementia were systematically distilled: (a)Comprehensive knowledge and attitudes of formal caregivers towards end-of-life care for dementia, (b) negative work experiences, (c) motivation to enhance care quality, and (d) unmet care needs. The results show that formal caregivers in China face significant physical and mental risks in end-of-life care for patients with advanced dementia, with their well-being levels fluctuating markedly compared to before employment. However, through long-term practice, adaptive coping strategies have been developed, including reshaping disease perceptions, establishing peer support systems, and redefining personal identity. Additionally, it has been determined that the needs of caregivers involve multiple levels, including operational process optimization at the micro level and organizational support at the meso level.

Aggressive behavior is defined as ‘non-accidental overt behavior involving the delivery of a harmful stimulus to an object, oneself, or any other person’ [32]. In the population of dementia patients, the incidence of verbal aggression is as high as 89%, while physical aggression and destructive behavior occur at rates of 61% and 25%, respectively. Notably, 67% of individuals who exhibit verbal aggression continue this behavior until the end stage of life. Physical aggression, on the other hand, shows self-limiting characteristics. Research analysis indicates that intimate care is considered the primary factor in triggering aggressive behavior, with hallucinations and delusions being secondary triggers [33]. The high frequency of aggression, which not only results in physical harm to caregivers but also induces long-term psychological stress (as reported by all caregivers in this study). This may be due to a reduced awareness of the environment caused by cognitive impairment, which in turn leads to intimate care being perceived as a threat. The present study’s identification of aggression as a surrogate for communication—such as the physical resistance described by L9—aligns with findings by M. Uri Wolf et al. [34] on aggravated behavior in dementia. However, coping strategies for immediate memory loss differed from those reported by Maria Cotell et al. [35], who emphasized the role of technological interventions, particularly non-invasive brain stimulation techniques. In contrast, findings from this study indicated that caregivers primarily relied on repetitive explanations as a coping strategy. This difference may be related to the allocation of nursing home resources, providing a new perspective on understanding the factors influencing strategies. However, the current results have not fully addressed the question of ‘how to effectively integrate strategies to comprehensively improve nursing outcomes.’ Future multi-center studies could explore the specific mechanisms linking resource allocation with the selection of nursing strategies; randomized controlled trials could also assess the practical effects of targeted training programs and the integration of non-verbal cues into end-of-life care, to clarify the role of interventions in enhancing the capabilities of caregivers and better meeting the needs of terminally ill patients, thereby providing a more comprehensive answer to the research questions.

Research shows that formal caregivers are experiencing significant occupational stigma [36], and the social devaluation of elderly care work ——, such as viewing it as a “lowly job” ——, severely undermines their professional identity, a phenomenon particularly pronounced in East Asian cultural contexts. Therefore, some caregivers have explicitly expressed the need for more understanding and recognition from society. At the same time, this study has identified a contradictory phenomenon within the group of formal caregivers: the coexistence of social stigma and occupational resilience. Some formal caregivers construct unique value identities through intergenerational empathy, linking the current condition of their end-of-life care subjects to their own future possibilities, and develop cognitive frameworks that either acknowledge or deny such outcomes. This cognitive framework not only motivates them to learn about dementia-related knowledge but also reinforces a positive attitude toward end-of-life care work. Moreover, knowledge accumulation positively influences end-of-life care practices, which is consistent with domestic research on the impact of BPSD knowledge on caregiving attitudes [37]. For the sustainable development of the elderly end-of-life care service system, ensuring the well-being of formal end-of-life care personnel in nursing homes and providing them with adequate psychological, material, and social support is crucial. Future research could focus on the characteristics of East Asian culture, delve into the impact of external environmental factors on the occupational identity, perceived social support, and practical behaviors of end-of-life care personnel, and develop end-of-life care career development and support strategies that align with local culture. Solving these problems can not only improve the job satisfaction and professional identity of end-of-life care workers, but also directly promote the overall quality of end-of-life care, and ultimately enable patients to benefit from better and more humanistic care services.

In the context of end-of-life care, structural human resource shortages and inadequate auxiliary equipment lead to overburdened long-term care, posing a direct and severe challenge to the quality of care for terminally ill patients. Studies have reported that the nurse-to-patient ratio during night shifts is 1:18, and during day shifts, it is 1:8, far exceeding the optimal ratio of 1:4 to 1:3 achieved after Japan implemented its Long-Term Care Insurance (LTCI) system, which aims to address the needs of an aging population [38]. Such resource shortages are even more pronounced in end-of-life care settings, where caregivers struggle to provide adequate emotional support and meticulous care for terminally ill patients. This study found that caregivers in nursing homes alleviate resource shortages through informal collaboration, although this has temporarily reduced work pressure, it is only a temporary solution and not a sustainable strategy. From the perspective of research questions, the current findings reveal the status quo and temporary coping methods of nursing resource shortages in end-of-life care, partially addressing the issue of “how to optimize the allocation of end-of-life care resources,” but have yet to fully resolve the construction of sustainable strategies. To delve deeper, future research could focus on: first, evaluating the actual effectiveness of dynamic personnel adjustments based on activities of daily living (ADL) scores using an “intelligent scheduling system” in end-of-life care scenarios; second, investigating the specific impacts of purchasing nursing assistive devices on reducing the physical burden on caregivers, improving the efficiency and quality of end-of-life care, and how these measures can be effectively integrated into the end-of-life care system to form long-term sustainable solutions. Through such research, we can more comprehensively answer the research questions and genuinely enhance the overall level of end-of-life care.

In the context of end-of-life care, the conflicts faced by caregivers profoundly reveal the complexity of structural contradictions. The notion that family members view caregivers as “tools” is particularly evident during the final stages of life —— As patients approach the end of their lives, family members often find themselves in a state of intertwined grief and stress, which intensifies the emotional detachment of caregivers, possibly reflecting the generational impact of the “four-two-one family” structure under China’s one-child policy [39]. For example, some family members regard “rescue at all costs” as a manifestation of filial piety, resulting in terminally ill patients being forced to suffer from excessive medical pain. The primary triggers for interpersonal conflicts stem from complex work environments; caregivers in end-of-life care not only bear the direct responsibility of caring for terminally ill elderly individuals but also serve as mediators to resolve conflicts among family members. This aligns with J. W. Getzels and E.G.Guba’ role expectation conflict theory [40]. From a research perspective, existing findings partially elucidate the role dilemmas and resource management conflicts faced by caregivers in end-of-life care, providing preliminary answers to the question of “how to optimize interpersonal interactions and resource allocation in end-of-life care,” though there is still room for further exploration. Future studies could investigate the impact of “de-tooling” educational interventions on changes in family members ‘attitudes during the final stage, analyzing whether such interventions can enhance family members’ understanding and respect for the caregiver’s role. In addition, the resource management dilemma in hospice settings manifests as fragmented authority, which aligns with Ostrom’s view that inadequate coordination of multi-center governance structures leads to conflicts [41]. The “relative deprivation” effect in welfare distribution also negatively impacts the enthusiasm of caregivers, as the positive emotional needs of formal caregivers in hospice environments must be reflected elsewhere. To improve this situation, management should establish clear policies for resource allocation standards and implement fair welfare distribution mechanisms. Further research could focus on the specific implementation paths of these policies in hospice settings, as well as their long-term effects on caregiver engagement, job satisfaction, and the quality of end-of-life care. Hospice care, as a specific form of end-of-life care, provides a more specific answer, optimizes the hospice care environment, and provides more targeted strategic support for end-of-life practice.

In the context of end-of-life care, this study found that the intrinsic motivation of formal caregivers in nursing homes exhibits intergenerational projection characteristics. Specifically, this is manifested through maintaining an optimistic attitude, fostering a strong sense of professional responsibility, and linking the current condition of the dying patient with their own aging and anticipated end-of-life experiences, thereby reinforcing positive values. This cognitive framework aligns with the value-driven theory proposed by Brook et al. within the VIPS framework [42]. Notably, in this special setting of end-of-life care, caregivers must not only address the deterioration of the patient’s physical condition but also handle the complex emotional support needs during this critical phase. At such times, the sustainability of intrinsic motivation is crucial for maintaining high-quality care, which heavily depends on the effectiveness of external support systems. Only when organizational training (such as specialized end-of-life care training) and material support (such as dedicated nursing plans for the end-of-life stage) reach adequate levels can intrinsic motivation be effectively transformed into sustained service efficiency, meeting the needs of dying patients and their families. To enhance resource management, it is recommended to develop intelligent material management systems using IoT technology, combined with the predictive replenishment algorithm proposed by Md Abrar Jahin et al. [43]. This approach will facilitate a paradigm shift from passive response to proactive prevention. However, in the current hospital environment, especially in end-of-life care units, implementing this system faces unique challenges —— end-of-life care often has limited resources and special needs, requiring a balance between cost and patient-specific demands, and careful consideration of feasibility and resource constraints.

It is worth noting that in the context of nursing homes in China, formal caregivers are constrained by their educational level and bear heavy work pressure (on average, one caregiver has to care for 8–10 elderly individuals with dementia at different stages and non-dementia seniors, leading to a “multi-role” situation). This makes it difficult for them to fully separate the mixed care content across different disease stages, resulting in practical confusion between end-of-life care and routine care. Although this study ensures that all participants have experience caring for terminally ill patients with late-stage dementia and strives to delve into elements related to end-of-life care from their narratives, the weak cultural foundation remains a prominent challenge faced by this group, which is also an issue that urgently needs improvement in China’s nursing home system. This situation profoundly highlights the urgency of strengthening cultural training and specialized end-of-life training: cultural training can enhance caregivers’ ability to absorb concepts and knowledge about end-of-life care, while specialized end-of-life training can specifically develop their professional skills, helping them accurately distinguish between end-of-life and routine care, thereby improving the professionalism and precision of end-of-life care services.

Converting the obstacles, facilitators, and actual needs faced by end-of-life care in the aging process into necessary prerequisites is a key cornerstone for building an effective end-of-life care system. Although existing research has provided insights into elderly care, there is still a lack of in-depth exploration of end-of-life care. A comprehensive analysis of the end-of-life care system requires extensive research, covering the challenges faced by formal caregivers in end-of-life care and their adaptation to cultural and economic differences across regions. It also involves evaluating the effectiveness of various management models in end-of-life scenarios, ensuring that samples cover diverse end-of-life care settings. Additionally, it is essential to investigate the impact of emerging technologies on end-of-life care services, particularly how advancements in smart devices and telemedicine enhance accessibility and professionalism in end-of-life services, ensuring that terminally ill patients receive more precise care. Exploring the synergistic mechanisms between end-of-life care services and other social services (such as medical and community services) is equally critical. It is necessary to clarify how end-of-life care can be seamlessly integrated into medical and community service networks to provide comprehensive support for terminally ill patients. These studies will optimize the end-of-life care system from multiple dimensions, promoting its development in a scientific and comprehensive manner, ultimately improving the quality of life during the final stages of life, allowing terminally ill patients to experience warmer and more professional care in their limited time.

### Study limitations

The study has certain limitations. The geographical scope is limited to Dongguan City, Guangdong Province, China, where the development level of hospice care, resource allocation, and cultural awareness may have regional characteristics. This limits the general applicability of the research findings to formal caregivers in nursing homes across the country (especially those in regions with different levels of economic and cultural development). Furthermore, the sample size is relatively small, making it difficult to delve into the rich experiences and challenges faced by caregivers in responding to the special needs of terminally ill patients and managing the complex psychological and physiological changes during the dying phase. This restricts the presentation of a comprehensive picture of hospice care. Due to the working environment and role characteristics of formal caregivers in China, they find it challenging to clearly differentiate between the care stages of dementia and end-of-life care. This ambiguity blurs the boundaries between end-of-life care and daily care. Consequently, even when end-of-life care is involved, formal caregivers often struggle to recognize and accurately communicate these aspects due to educational limitations, as highlighted in the results section of the article. This situation severely hinders further research in this area. During data collection and analysis, personal subjective factors of researchers are more likely to interfere when dealing with highly emotional and value-laden content related to hospice care, thereby affecting the objectivity of the results.

## Conclusion

In summary, the end-of-life care for patients with advanced dementia is a complex and multidimensional process involving close interactions among patients, caregivers, and social support systems. Due to cognitive decline, patients often exhibit abnormal behaviors and functional declines; therefore, accurately identifying their potential needs in the terminal stage is crucial for effective care. Formal caregivers, as the primary providers of end-of-life care for patients with advanced dementia, are significantly influenced by working conditions that markedly impact the quality of end-of-life care. The long-term high workload leads to occupational stress, compounded by negative emotions conveyed during the late stages of illness, placing most formal caregivers in a negative work environment.In this study, the negative working conditions and environments faced by formal caregivers overshadow the real core challenges of end-of-life care for patients with late-stage dementia in practical settings. Therefore, improving the basic working conditions of formal caregivers and minimizing negative environmental factors are crucial to overcoming current practical challenges and systematically enhancing the quality of end-of-life care for patients with late-stage dementia. This highlights the necessity of targeted interventions. A well-structured social support system is equally critical; optimizing human resource allocation, training resources, and the distribution of necessary caregiving materials can both alleviate the burden on caregivers and improve the quality of end-of-life care. Future research should focus on integrating these interventions into routine end-of-life care practices to provide better life-end stage care for patients with advanced dementia. This direction holds significant guiding importance for deepening end-of-life care research and optimizing clinical practice.

## Electronic supplementary material

Below is the link to the electronic supplementary material.

Supplementary Material 1

## Data Availability

Upon reasonable request, interview recordings or transcript data can be obtained from the corresponding author. To protect participant confidentiality, personal identifiers have been replaced with participant numbers (e.g., Participant 1, Participant 2).
